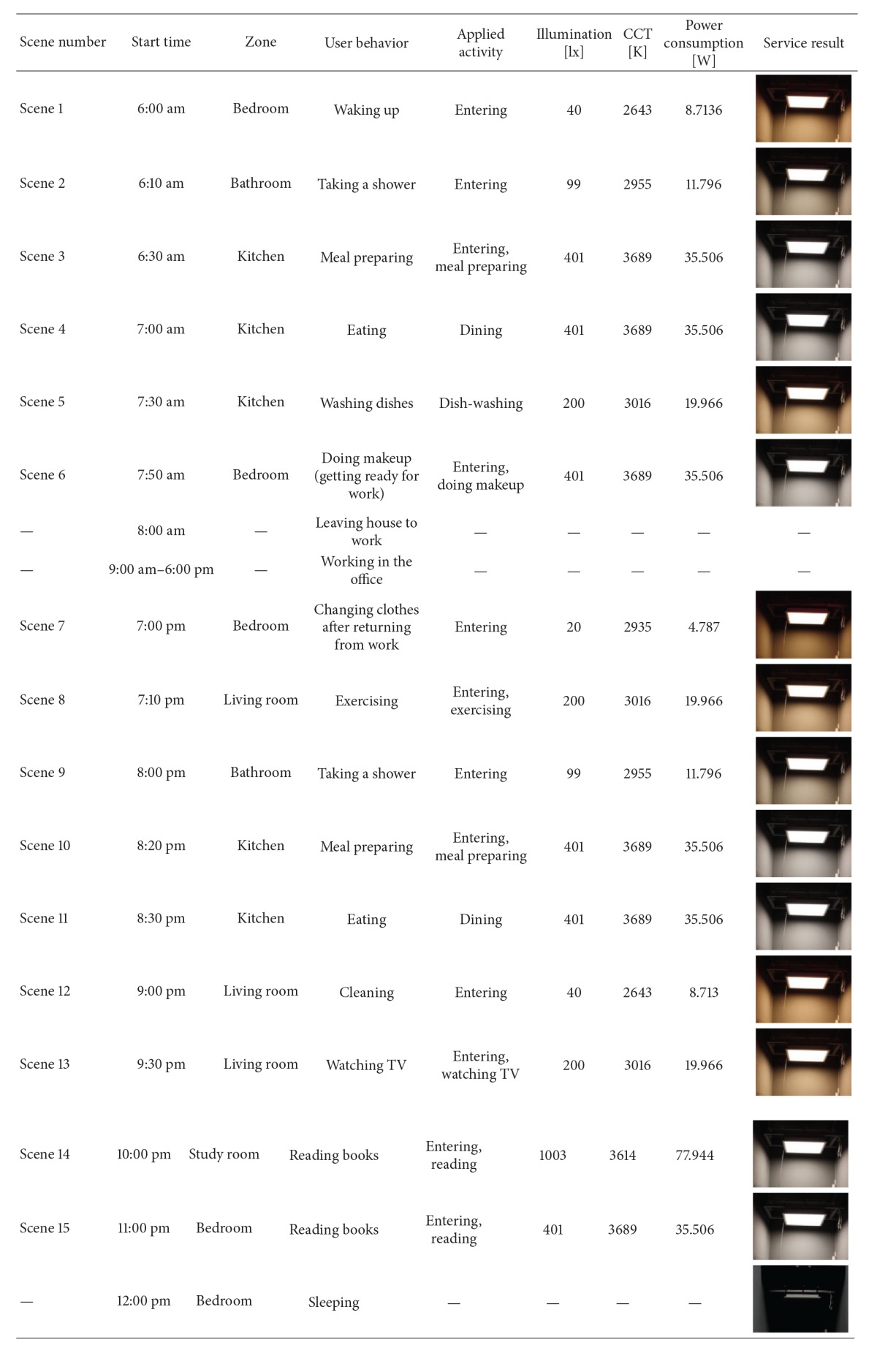# LED Context Lighting System in Residential Areas

**DOI:** 10.1155/2014/851930

**Published:** 2014-06-29

**Authors:** Sook-Youn Kwon, Kyoung-Mi Im, Jae-Hyun Lim

**Affiliations:** ^1^Green Energy Technology Research Center, Kongju National University, Cheonan 331-717, Republic of Korea; ^2^Department of Computer Science and Engineering, Kongju National University, Cheonan 331-717, Republic of Korea

## Abstract

As issues of environment and energy draw keen interest around the globe due to such problems as global warming and the energy crisis, LED with high optical efficiency is brought to the fore as the next generation lighting. In addition, as the national income level gets higher and life expectancy is extended, interest in the enhancement of life quality is increasing. Accordingly, the trend of lightings is changing from mere adjustment of light intensity to system lighting in order to enhance the quality of one's life as well as reduce energy consumption. Thus, this study aims to design LED context lighting system that automatically recognizes the location and acts of a user in residential areas and creates an appropriate lighting environment. The proposed system designed in this study includes three types of processing: first, the creation of a lighting environment index suitable for the user's surroundings and lighting control scenarios and second, it measures and analyzes the optical characteristics that change depending on the dimming control of lighting and applies them to the index. Lastly, it adopts PIR, piezoelectric, and power sensor to grasp the location and acts of the user and create a lighting environment suitable for the current context.

## 1. Introduction

As such issues as global warming, energy crisis, and atomic energy plant stability in the face of earthquakes and tsunami draw attention, interest in environmental and energy issues rapidly increases around the globe [1–3]. In addition, as concerns in well-being and quality of life continue to rise, the demands among users involving lightings gradually advance from mere brightness and efficiency to the quality of lighting environment. Accordingly, LED digital control convergence technology, which replaces low-efficiency light bulbs and fluorescent light containing harmful substances such as mercury, has led to the creation and increasing popularity of emotional and human-friendly lighting environment [3–6].

LED is advantageous in terms of high efficiency, low-power consumption, long lifespan, and environment-friendliness. In addition, it can make use of a broad range of wavelengths of light, including visible rays and, thus, is regarded as the next generation lighting through which a variety of lighting settings can be created [7]. It goes beyond the function of illuminating and utilizes various features of light that affect the visual and nonvisual experiences of humans [4]. Recently, this type of LED lighting has advanced into a smart system lighting that reflects the emotions of users, pursues convenience in daily life, and saves energy in convergence of IT and knowledge of various elements such as surrounding environments, human behaviors, and so forth [6, 8]. Most of the existing lighting systems, however, focus on improving energy efficiency by automatically turning the light on or off depending on the user's presence or location [9, 10] or controlling dimming function depending on the amount of natural light coming in [11–13]. Further, when the color temperature is to be adjusted depending on the user's emotional state, direct control by means of controlling devices such as remote controller is required, which causes inconvenience [14]. Hence, it is necessary to develop a futuristic lighting system that recognizes the user's emotional state, location, and activity by means of sensors; provides the optimal lighting environment accordingly; guarantees convenience and comfort; and enhances the energy consumption ratio as well. To develop such a system, it is necessary to utilize IT-based means such as context sensing technology, lighting control technology for the precise adjustment of the luminous source, interlighting communication technology, software, and so forth [10, 15].

Among major controlling elements to save energy and provide the user with a sense of comfort are illuminance and color temperature [16]. When the lighting environment in the space where the user resides does not reach the targeted illuminance, it may decrease working efficiency, worsen eyesight, or cause anxiety, mental depression, nervousness, and various other disorders. In contrast, when it exceeds the targeted illuminance, the difference in brightness may cause dazzle reflex and wasting of energy. Hence, the appropriate level of illuminance needs to be secured in full consideration of spatial characteristics and user behavior. When the same level of intensity is maintained in a space, keeping the color temperature high above 6,000 K will enhance the concentration while keeping it below 3,000 K enhances a sense of comfort. Hence, the proper color temperature also needs to be taken into consideration depending on the user's working condition [6].

Two of the currently available standards for illuminance are KS A 3011, which is the domestic standard of KS, and the standard recommended by IESNA in North America, defined depending on the types of user activities [17]. Neither of the two standards, however, presents a standard on the range of color temperature except experimental researches on the influence of the combination of illuminance and color temperature on human emotions [5]. Hence, it is necessary to design the standard and index of illuminance and color temperature to provide lighting environments appropriate for the user's location and behavior.

Accordingly, this study aims to suggest LED context lighting system that designs contextual lighting environment indexes, automatically recognizes user location and behavior, and creates lighting environments depending on the context. The performance then is evaluated by applying the system suggested according to the lighting control scenario. KS A 3011 is referred to as the standard for illuminance and lighting environment indexes and Kruithof's curve for color temperature [18]. To verify the effectiveness of the suggested system, such factors as illuminance, color temperature, and electric power of wWcW LED that change depending on the dimming control are measured and referred to as the basis for system performance evaluation.

This study consists of the following sections.  Section 2 grasps current problems on the basis of standards for spatial illuminance recommended by KS A 3011 and IESNA and the actual illuminance measures of 16 domestic residential areas. Examined are the kinds and characteristics of sensors for contextual recognition as well as Kruithof's curve illustrating the relevance between users' comfort and the factors of illuminance and color temperature.  Section 3 designs the LED context lighting system suggested in this study and defines the lighting environment indexes and control scenarios.  Section 4 measures and analyzes optical characteristics and power data depending on the dimming control of LED lighting and then examines the contextual interpretation process for the recognition of a user's location and behavior. In addition, the suggested system is compared with existing lighting environments according to their lighting control scenarios and in terms of comfort and energy efficiency in order to evaluate the suggested system's performance.  Section 5 presents the conclusion and future direction of the study.

## 2. Related Work

When it comes to creating appropriate lighting environments in consideration of the user's current context, the basic standard to be referred to is required for the control of illuminance, color temperature, and so forth.  Table 1 shows an example of illuminance standards currently recommended in Korea and the U.S. that were defined in reflection of the characteristics of each residential space and user behavior [19, 20].

**Figure 1:**
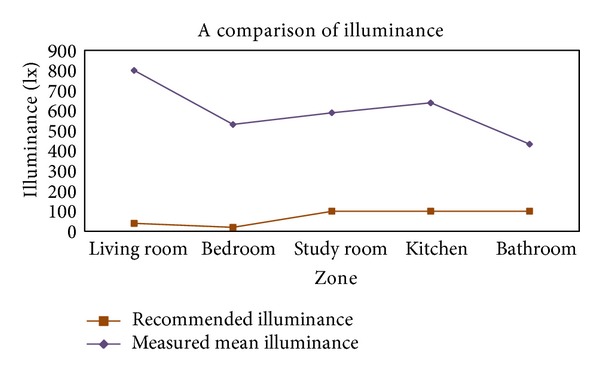
A comparison between recommended illuminance and measured mean illuminance in residential areas.

The domestic standard of KS for illuminance specifies the scope of illuminance: min.–mid.–max. The standard of IESNA, U.S., includes more detailed factors such as spatial context and feature, user age, work speed/accuracy, and so forth, based on the scope of illuminance stated above. According to one domestic research [21] that measured the average illuminance in each zone of residential space, the average illuminance of different places exceeded the illuminance standard of KS as shown in Figure 1, which indicated that the waste of energy for lighting was serious. According to the same research, this was because of the general tendency of Koreans to prefer bright illumination regardless of work types in the space. Hence, this study aims to provide users with a sense of comfort as well as enhance working and energy efficiency by creating lighting environments according to work types.

**Figure 2:**
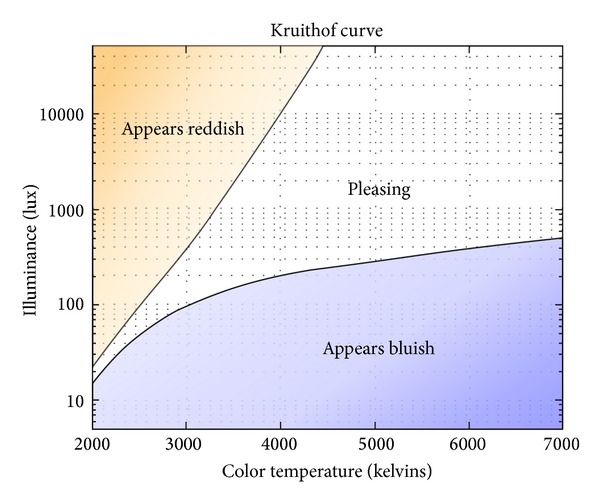
Kruithof's comfort curve.

In 1941, Kruithof, a scientist in Holland, illustrated the relevance between human comfort and the combination of brightness and color temperature with the Kruithof's comfort curve as shown in Figure 2. This has been utilized as the control index of artificial lighting systems. The horizontal axis in the graph indicates the correlated color temperature [K] while the vertical axis illuminance E[lx] increases at a log rate log of 10 lx. Two curves are drawn on the diagram. They show the limits between an illumination considered “pleasing” or not. Kruithof reported that beneath the lower illuminance limit, illumination is judged as “dim” at low CCT and “cool” at high CCT. Above the upper illuminance limit, color reproduction is unnatural and unpleasant [18]. To create lighting environments in reflection of user location and behavior, this study designs the lighting environment indexes in utilization of the illuminance standard in Table 1 and the area of “pleasing” in Figure 2.

Existing lighting control systems take advantage of sensor technology to recognize a user's presence as a controlling strategy to reduce energy consumption.  Table 2 compares the performances of different sensors, among which are PIR, ultrasonic, and microwave, which are used to recognize a user's presence. In Table 2, “resolution” shows the result of comparatively analyzing each sensor's performance in terms of user presence and number, location, initial cost, and so forth. It indicates that the resolution of video cameras or bionics recognition systems is superior to that of other sensors. Such sensors may not be preferred for such considerations as high prices and lack of privacy, but they are advantageous in locating a person in case of dangerous contexts such as fire. Recently, a hybrid type of sensor that combines two sensors such as PIR/ultrasonic and PIR/microwave has been mainly used to make up for each sensor's disadvantages and enhance recognition rates.

Existing automated lighting control systems recognize user locations and surrounding illuminance sources by means of human presence sensors and illuminance sensors for energy saving and adopt the dimming control methods. However, they do not create lighting environments in recognition of user behavior. In the case of color temperature adjustable systems, the user should adjust the lighting environments manually by manipulating such means as smart phone application, remote controller, and so forth. To solve this problem, this study designs LED context lighting system that automatically recognizes contextual changes by means of various sensors and controls lighting environments such as illuminance, color temperature, and light on/off automatically.

## 3. LED Context Lighting System

### 3.1. Design of Context Lighting System

**Figure 3:**
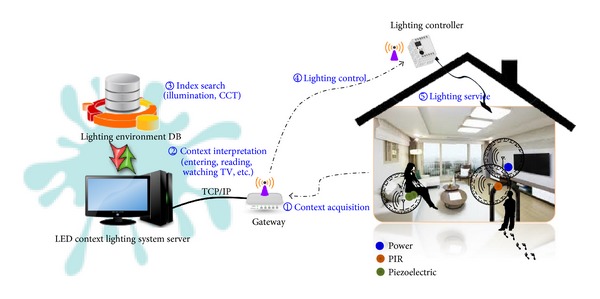
Conceptual diagram of LED context lighting system.

The structure of LED context lighting system suggested in this study is illustrated in Figure 3.

The suggested system involves the five steps of processing as shown in Figure 3. Context acquisition module collects sensing data periodically from the PIR, piezoelectric, and power sensors to recognize user location and behavior. PIR is a sensor of infrared light waves and heat generated from a human body, which are utilized to grasp a user's presence in a certain area of the residential space. A piezoelectric sensor is a sensor that converts vibration into electric signals that is used to trace a user's movement and behavior effectively. A power sensor is used to recognize the user's behavior and alterations in power consumption in linkage with other electric appliances. The sensing data collected from sensors go through the gateway and is transmitted to the LED context lighting system server as input data for user context inference.  Table 3 shows the combination of sensors that recognizes various contexts (location, behavior) in five different zones of the residential area [22]. For example, one or more PIR sensors are used at the entrance to recognize a person's staying in or leaving and the number of people in the room. In addition, when a user is watching TV while sitting on the sofa in the living room, a piezoelectric sensor and a power sensor, installed on the sofa and TV set, function to sense whether the user is sitting or turning the TV on/off.

To create lighting environments suitable for the inferred user context, the lighting environment indexes saved in the database (illuminance, color temperature) and the LED dimming control rates are referred to. Lastly, transmission packets for lighting control are generated and sent to the controller through the gateway for a lighting environment appropriate for the user's current context. PLC, ZigBee, Bluetooth, Wi-Fi, and so forth are commonly used when it comes to operating a home area network (HAN). However, Bluetooth and Wi-Fi communication are disadvantageous in terms of protocol complexity, cost, and power consumption; thus, wire-based PLC or wireless ZigBee is preferable [23]. In particular, ZigBee/IEEE 802.15.4 is one of the major wireless sensor network technologies that feature low-power consumption, low price, and convenient use. This standardizes the upper protocol and application based on the PHY/MAC layer specified by the committee of IEEE 802.15.4 in 2003 [24–26]. The system suggested in this study adopts ZigBee wireless communication protocol and TCP/IP protocol. The sensor and gateway emphasize mobility and energy efficiency by transmitting data through ZigBee protocol while the gateway and server are designed for remote control by means of TCP/IP protocol.

### 3.2. Design of Lighting Environment Indexes and Control Scenario

Table 4 defines the lighting environment (illuminance, color temperature) indexes depending on user location and behavior. Among the indexes, illuminance corresponds to the “middle illuminance” standard according to KS A 3011, the Korean illuminance standard, while the range of color temperature corresponds to the area of “pleasing” based on the illuminance standard of Kruithof's curve. Kruithof's graph presents the relevance between the user's comfort and the combination of illuminance and color temperature as follows: the lower section of illuminance down to 50–100 lx involves color temperature as low as 2,500 K–3,000 K while the higher section up to 500 lx corresponds to color temperature as high as 3,000 K. According to the designed lighting environment indexes as shown in Table 4, the low section of illuminance down to 20–100 lx involves color temperature ranging from 2,000 to 2,800 K, the section between 200 and 400 lx about 2,700–4,500 K, and 1,000 lx about 3,500 K or higher.

**Figure 4:**
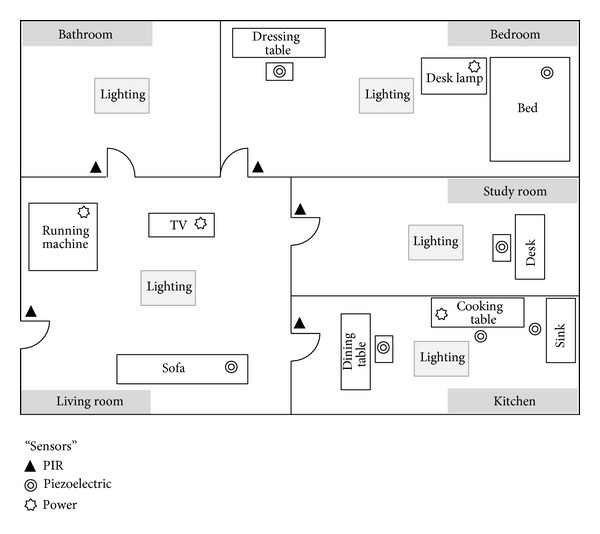
A virtual residential area.

Figure 4 shows a virtual residential area that is divided into five zones—bedroom, living room, study room, kitchen, and bathroom—for the performance evaluation of the suggested system. Each zone has various sensors for contextual recognition, lighting fixtures, and electric appliances. The lighting control scenario was designed to adjust the illuminance and color temperature as in Table 5 depending on the residents' behaviors and movement patterns in each time of the day. The scenario supposes that lightings are used in the residential area for 7 hours in total and that there could be 15 different contexts except “sleeping” during which the user does not stay in the zone or use lightings.

## 4. Experiment and Evaluation

### 4.1. Experiment and Analysis of Optical Characteristics with Dimming under Control

**Figure 5:**
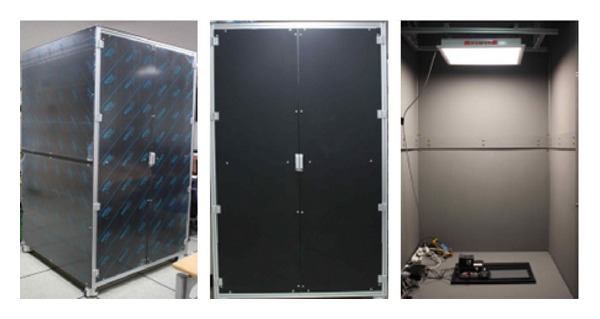
A black lighting box (side, front, inside).

**Figure 6:**
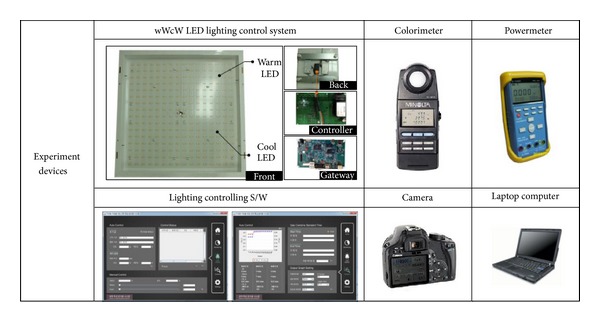
Automatic logging system for optical characteristic measurement.

This study includes an experiment with the following procedures to collect basic data necessary for lighting environment indexes. First of all, a black lighting box (W/L/H: 1.28 m, 1.28 m, 2.08 m) coated with N5 substance was used as the experiment space. The object lighting was wWcW LED, which included 256 units of low color temperature light sources down to 2,800 K (warm white) and high color temperature light sources up to 6,500 K (cool white). The used lighting supported 256 steps of dimming control by means of the wireless lighting controller. As five steps were adjustable at a time, the output range was 0–1089 lx in the case of illuminance and 2,635–5,718 K in the case of color temperature. The black lighting box used as the experiment space is shown in Figure 5.  Figure 6 shows the automatic logging system that contained such elements as wWcW LED lighting, lighting controller, gateway for data transmission between the server and controller, lighting controlling S/W, colorimeter of CL-200 A model to measure the optical characteristics such as illuminance and color temperature, electric power meter (HPM-300A), digital camera (Canon 450D) for photographing, server (laptop computer), and so forth.

**Figure 7:**
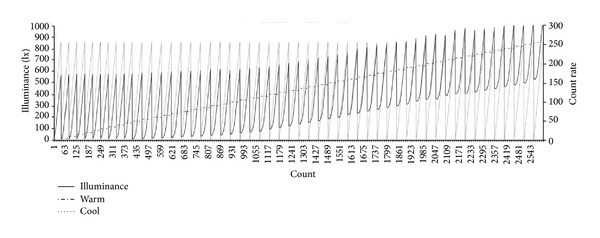
Changes in illuminance depending on dimming control.

**Figure 8:**
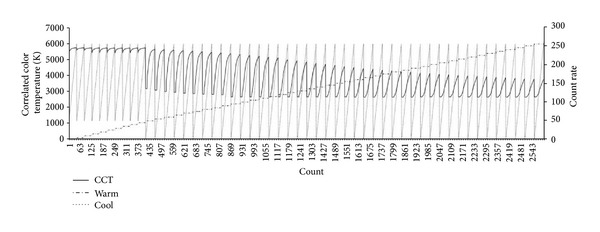
Changes in color temperature depending on dimming control.

The experiment procedures for lighting environment index extraction are as follows. First, a wWcW LED lighting fixture with a controller embedded is installed 1.55 m from the ground. The level in the black lighting box is to be maintained. In general, the ceiling height of a residential space is 2.4 m, and the working plane is 0.85 m high. These figures are applied to the experimental environment. Second, a colorimeter is installed in the center of the ground toward which the lighting device generates the light vertically; additionally, the electric meter and camera are installed at the proper locations to collect the basic data including optical characteristics such as illuminance and color temperature, changes in power consumption, and so forth. To collect such data automatically, this study uses Java language for an illumination-controlling application, through which the dimming levels of 256 steps increase 5 steps at a time and LED lighting is controlled at every 20 seconds by way of pulse width modulation (PWM). The data of optical characteristics and electric power that may change depending on the lighting control are processed collectively, saved in the database, and utilized as the basis for lighting environment index generation. Figures 7 and 8 show the result of dimming in combination with the warm and cool LED light source control rates.  Figure 7 indicates that the range of illuminance control is 0–1089 lx while Figure 8 indicates that the range of color temperature is 2,635–5,718 K.

Lastly, the actual measurements that corresponded to the lighting environment indexes in Table 4 were extracted in utilization of the integrated database, and the results are presented in Table 6. Table 6 shows factors based on the lighting environment indexes such as illuminance, color temperature, tristimulus values (*XYZ*), power consumption, dimming control rates of different light sources, sum of control rates, and so forth. As shown in the result, illuminance and power consumption were in lineal proportion to each other. When the deviation value of control rates among light sources was small, color temperature gradually increased up to 4,000 K. In contrast, when the deviation values of light sources including the warm white LED light source were large, the color temperature went down to 2,600 K.

### 4.2. Contextual Recognition and Service in Reflection of User Location and Behavior

This chapter examines the contextual recognition process to accurately grasp contexts such as current user location and behavior by means of wireless communication network and sensors. Context information is about the user's environment and can be collected by means of the sensors installed in the space. The process of grasping the situation is called “context awareness,” and this enables providing environmental control service according to individuals' demands. In utilization of such context awareness, context awareness system that provides users with service according to their needs requires such techniques as context acquisition and context reasoning. Situational information collected by means of the context acquisition module is divided into the low-level context and high-level context. Once the low-level context information is collected from sensors or computing devices, it is converted to high-level context information through various processing steps such as inference and then utilized for service.

**Figure 9:**
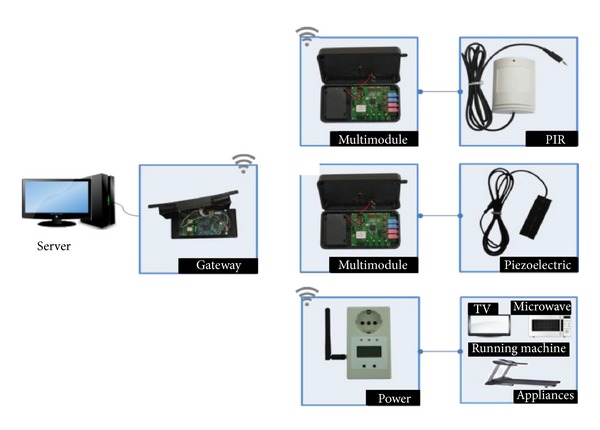
Types of context awareness sensors and a gateway.

As shown in Figure 9, the suggested system senses user location, behavior, and power consumption by means of the three types of sensors (PIR sensor, piezoelectric sensor, and power sensor) for context acquisition, and then the collected data is sent to the server through the gateway as information for context awareness. The power sensor is connected directly to home appliances while the PIR and piezoelectric sensors were designed to be connected to a multimodule for a combination of multiple sensors. Data packets from each sensor are transmitted to the server through the gateway. A packet consists of 22 bytes as shown in Table 7. The module ID of the first byte in the entire data packet is for sensor recognition. The value of ID is decided depending on the sensor type. That of PIR sensor is 0x00-0x09, that of piezoelectric sensor is 0x1E-0x27, and that of power sensor is 0x28-0x3B, each of which means that it is possible to combine 10, 10, and 20 sensors, respectively. The data values from the PIR sensor and piezoelectric sensor are saved in data no. 0 of the 10th and 11th bytes. As the types and numbers of combined sensors vary and increase, the values are stored in data nos. 1–3. PIR sensors recognize user motions and save 0 or 1 in the corresponding byte area. The sensing data of the piezoelectric sensor indicates force (g) of the gravity acceleration that may change depending on voltage (V). The instantaneous electric power of home appliances and lighting fixtures recognized by the power sensor is saved in the area of byte nos. 10–13 while the accumulated value is saved in the area of byte nos. 14–17.

Table 8 shows examples of combining the module IDs of sensors installed to accurately grasp the user's zone-specific location and behavior. This information should be registered to the system in advance.

**Figure 10:**
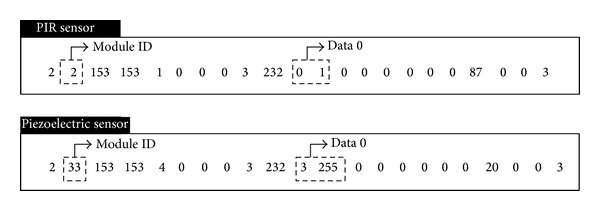
A packet of received data in the situation of “reading.”

The raw context data of data packets collected from sensors are converted into low-level context information as shown in Table 9 and then abstracted to high-level context information. For instance, when context elements include zone, location, pressure intensity, and power consumption and raw context data is *R*_*i*_ = {201, 33, 3255, 0}, the system converts them into low-level context information, LCi = {21, 33, 997 g, 0w}, according to the operation rule and in consideration of the hardware characteristics of each sensor. In the final stage, user location and behavior are interpreted in relation to study room, reading, and so forth.  Figure 10 shows the output of data packets received from sensors as the recognized situations.

Table 10 shows the structure of data packets to transmit control orders to the controller of wWcW LED for lighting environments appropriate for the current situation. Module ID of 0x64-0x96 that corresponds to the first byte of the data packet is necessary to identify each wWcW LED lighting. In this case, it means that 51 lightings in total can be combined. The information of R, G, B, warm, and cool control rates depending on the light source of LED is stored in the area of byte nos. 5–9. As wWcW LED is used in this experiment, the dimming control rates of warm white and cool white LED extracted on the basis of lighting environment indexes in Table 6 are stored in byte nos. 8 and 9 and then transmitted to the controller.

**Figure 11:**
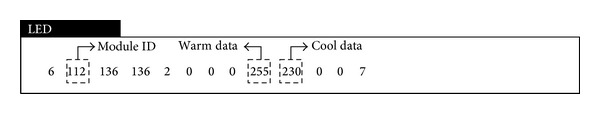
A transmission data packet for lighting control in the situation of “reading” and the service result.

Figure 11 shows the result of a transmission data packet output from the controller and lighting control service for the lighting environments of 1003 lx and 3614 K that corresponds to the user's act of “reading” in the “study room.”

### 4.3. Performance Evaluation of LED Context Lighting System

This chapter presents the result of restructuring lighting environments dynamically according to the scenario in Table 5 based on the lighting environment indexes in Table 6 for performance evaluation of the suggested LED context lighting system (Table 11).

**Figure 12:**
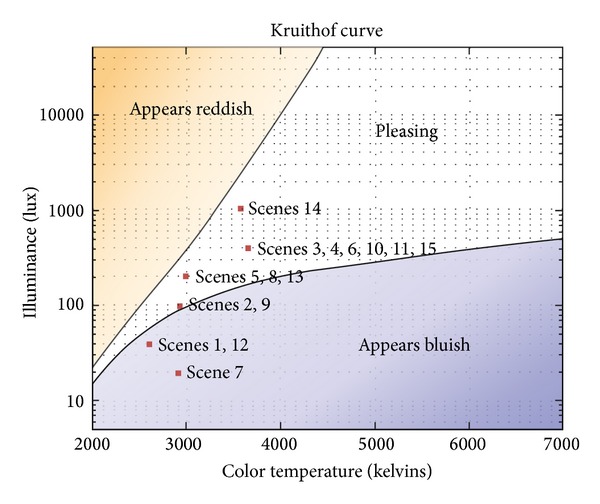
System performance evaluation in terms of comfort.

The performance evaluation of the system suggested in this study basically considers two factors: comfort and energy efficiency. First of all, to evaluate if the lighting environment secures a sense of comfort for the different locations and behaviors of the user, Figure 12 shows situational illuminance and color temperature levels in the form of Kruithof's curve. Due to the hardware limitation of wWcW LED used in the experiment, the lighting environments of scene 1 (6:00 am), scene 7 (7:00 pm), and scene 12 (9:00 pm), which corresponded to the targeted illuminance of 20 lx and 40 lx, were not included in the area of “pleasing,” but the other lighting environments reached the proper level of comfort.

**Figure 13:**
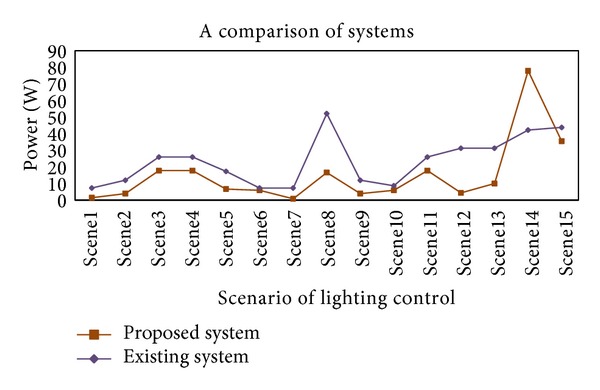
System performance evaluation in terms of energy saving.

In addition, this study comparatively analyzes lighting environments and the energy consumption of existing systems; the suggested one is shown in Figure 13 for system performance evaluation in terms of energy-saving effects. As for energy consumption in existing lighting environments, the average illuminance values in 17 domestic residential spaces were measured and presented in Figure 1. Afterwards they were examined based on the power consumption data collected for the experiment. According to Figure 13, existing lighting environments exceeded the recommended domestic upper limit of illuminance, and the consumption in each scene was higher than that of the suggested system except for scene 14. Scene 14 supposes that the user is “reading” in the “study room.” In existing lighting environments, the average illuminance value of the actual measurements in 17 study rooms was 589.76 lx. As the standard for illuminance defined in this study was 1000 lx, the saving effect of the suggested system calculated based on the deviation was 46%. In comparison with the lighting energy consumption in the scenario, existing lighting environments consumed 350.76 W while the suggested system consumed only 226.29 W, which indicated about 35% saving effect.

## 5. Conclusion

This study suggests LED context lighting system that automatically recognizes user location and behavior in a residential area and creates appropriate lighting environments. Existing LED control systems focus on energy consumption by way of automatically turning lights on or off and adjusting illuminance by sensing the user's location without considering motions and behaviors. In addition, systems that include the function of adjusting color temperature according to the user's emotional change involve inconvenience in manually manipulating controlling devices such as a remote controller. In contrast, the suggested system takes advantage of three sensors—PIR sensor, piezoelectric sensor, and power sensor—to automatically recognize a user's presence, sitting on a chair or sofa, or usage of home appliances in reference to changes in power consumption rates. This way, it interprets the current situation and creates lighting environments accordingly (illuminance and color temperature). This study classifies user situations in a residential area into (14 kinds (see Table 3) and designs situational lighting environment indexes (see Table 6) and lighting control scenarios (see Table 5)) by means of the domestic illuminance standard of KS and Kruithof's comfort curve. In addition, it actually measures and analyzes optical characteristics and power consumption that would change depending on the dimming control of wWcW LED, which are applied to the indexes and scenarios. The performance of the suggested system was evaluated based on two factors: comfort and energy efficiency. As a result, the lighting environments in all scenes, except scene 1, scene 7, and scene 12, which corresponded to the targeted level of illuminance (20 lx and 40 lx), were included in the area of “pleasing,” which indicated that the lighting environment provided the user with a sense of comfort. As a result of comparing the general lighting energy consumption in the scenarios, it turned out that the existing lighting environments consumed 350.76 W while the suggested system consumed only 226.29 W, indicating about 35% saving effect. In other words, LED context lighting system that reflects user behavior as well as location creates more convenient and comfortable lighting environments by dynamically restructuring lighting environments depending on the current situation. Additionally, energy consumption is reduced.

In the future, it is necessary to develop an algorithm to solve the problem of context conflicts when two or more users are present in the same zone. Further study should seek ways to reduce lighting energy consumption while taking into account a comfortable lighting environment and alternatively combining lighting devices and blinds during periods when natural light enters the indoor space.

## Figures and Tables

**Table 1:** Recommended level of illuminance (KS A 3011, IESNA).

Classification	Korea (KS) [lx]	U.S. (IESNA) [lx]
Living room		
General	30-40-60	50-75-100
Activity (family activity, entertainment)	150-200-300	50-75-100
Activity (reading)	300-400-600	200-300-500
Activity (exercise)	150-200-300	200-300-500
Bedroom		
General	15-20-30	50-75-100
Activity (makeup)	300-400-600	200-300-500
Activity (reading)	300-400-600	200-300-500
Study room		
General	60-100-150	50-75-100
Activity (reading)	600-1,000-1,500	500-750-1,000
Dining room		
General	60-100-150	50-75-100
Activity (dining table, kitchen table)	300-400-600	100-150-200
Activity (sink)	150-200-300	500-750-1,000
Bathroom		
General	60-100-150	50-75-100

**Table 2:** Comparison of current occupancy sensing technologies.

Type of sensor	Resolution	Number of occupants	Person identification	Person localization	Initial cost
PIR	Low	No	No	No	Low
Ultrasonic	Low	No	No	No	Low
Microwave	Low	No	No	No	Low
Sound	Low	No	No	No	Low
Light barriers	Low	Yes	No	No	Low
Video	Very high	Yes	Yes	Yes	High
Biometric	High	Yes	Yes	No	High
Piezoelectric	Low	No	No	No	Medium

**Table 3:** A context sensor that recognizes user location and behavior.

Zone	Location	Activity	Sensors		
			PIR	Piezoelectric	Power
Living room	Entrance	Entering	●		
	Sofa	Watching TV		●	●
		Reading		●	
	Treadmill	Exercising			●

Bedroom	Entrance	Entering	●		
	Bed	Reading		●	●
	Dressing table	Doing makeup		●	

Study room	Entrance	Entering	●		
	Reading chair	Reading		●	

Kitchen	Entrance	Entering	●		
	Kitchen chair	Dining		●	
	Cooking table	Meal preparing		●	●
	Sink	Dish-washing		●	

Bathroom	Entrance	Entering	●		

**Table 4:** Lighting environment indexes suitable for different user locations and behaviors.

Zone	Location	Activity	Illuminance [lx]	Color temperature [K]
Living room	Entrance	Entering	30-40-60	2,250–2,400
	Sofa	Watching TV	150-200-300	2,700–3,500
		Reading	300-400-600	3,000–4,500
	Treadmill	Exercising	150-200-300	2,700–3,500

Bedroom	Entrance	Entering	15-20-30	2,000
	Bed	Reading	300-400-600	3,000–4,500
	Dressing table	Doing makeup	300-400-600	3,000–4,500

Study room	Entrance	Entering	60-100-150	2,500–2,800
	Reading chair	Reading	600-1,000-1,500	more than 3,500

Kitchen	Entrance	Entering	60-100-150	2,500–2,800
	Kitchen chair	Dining	300-400-600	3,000–4,500
	Cooking table	Meal preparing	300-400-600	3,000–4,500
	Sink	Dish-washing	150-200-300	2,700–3,500

Bathroom	Entrance	Entering	60-100-150	2,500–2,800

**Table 5:** A lighting control scenario.

Start Time	User behavior	Applied activity	Living room		Bedroom		Study room		Kitchen		Bathroom	
			lx	K	lx	K	lx	K	lx	K	lx	K
6:00 am	Waking up	Entering	—	—	20	2,000	—	—	—	—	—	—
6:10 am	Taking a shower	Entering	—	—	—	—	—	—	—	—	100	2,500–2,800
6:30 am	Meal preparing	Entering, meal preparing	—	—	—	—	—	—	400	3,000–4,500	—	—
7:00 am	Eating	Dining	—	—	—	—	—	—	400	3,000–4,500	—	—
7:30 am	Washing dishes	Dish-washing	—	—	—	—	—	—	200	2,700–3,500	—	—
7:50 am	Doing makeup (getting ready for work)	Entering, doing makeup	—	—	400	3,000–4,500	—	—	—	—	—	—
8:00 am	Leaving house to work	—	—	—	—	—	—	—	—	—	—	—
9:00 am–6:00 pm	Working in the office	—	—	—	—	—	—	—	—	—	—	—
7:00 pm	Changing clothes after returning from work	Entering	—	—	20	2,000	—	—	—	—	—	—
7:10 pm	Exercising	Entering, exercising	200	2,700–3,500	—	—	—	—	—	—	—	—
8:00 pm	Taking a shower	Entering	—	—	—	—	—	—	—	—	100	2,500–2,800
8:20 pm	Meal preparing	Entering, meal preparing	—	—	—	—	—	—	400	3,000–4,500	—	—
8:30 pm	Eating	Dining	—	—	—	—	—	—	400	3,000–4,500	—	—
9:00 pm	Cleaning	Entering	40	2,250–2,400	—	—	—	—	—	—	—	—
9:30 pm	Watching TV	Entering, watching TV	200	2,700–3,500	—	—	—	—	—	—	—	—
10:00 pm	Reading books	Entering, reading	—	—	—	—	1,000	from 3,500 to more than 3,500	—	—	—	—
11:00 pm	Reading books	Entering, reading	—	—	400	3,000–4,500	—	—	—	—	—	—
12:00 am	Sleeping	—	—	—	—	—	—	—	—	—	—	—

**Table 6:** Actual measurements of optical characteristics and power consumption that correspond to the lighting environment indexes.

Zone	Location	Activity	Illuminance [lx]	Color temperature [K]	*X*	*Y*	*Z*	Warm control rate	Cool control rate	Power consumption
Living room	Entrance	Entering	40	2,643	63.87522	57.22073	14.25261	100	0	8.7136
	Sofa	Watching TV	200	3,016	216.8744	200.4531	81.37658	140	90	20.511
		Reading	401	3,689	416.6726	401.0262	243.5176	155	150	35.506
	Treadmill	Exercising	200	3,016	216.8744	200.4531	81.37658	140	90	20.511

Bedroom	Entrance	Entering	20	2,935	24.25033	22.37136	8.072989	65	25	4.787889
	Bed	Reading	401	3,689	416.6726	401.0262	243.5176	155	150	35.506
	Dressing table	Doing makeup	401	3,689	416.6726	401.0262	243.5176	155	150	35.506

Study room	Entrance	Entering	99	2,955	107.7683	99.20961	37.88805	115	65	11.796
	Reading chair	Reading	1,003	3,614	1050.55	1,003.439	601.261	255	230	77.94444

Kitchen	Entrance	Entering	99	2,955	107.7683	99.20961	37.88805	115	65	11.796
	Kitchen chair	Dining	401	3,689	416.6726	401.0262	243.5176	155	150	35.506
	Cooking table	Meal preparing	401	3,689	416.6726	401.0262	243.5176	155	150	35.506
	Sink	Dish-washing	200	3,016	216.8744	200.4531	81.37658	140	90	20.511

Bathroom	Entrance	Entering	99	2,955	107.7683	99.20961	37.88805	115	65	11.796

**Table:** (a)

0	1	2~3	4	5	6~9

STX (1 byte)	Module ID (1 byte)	Gateway ID (2 bytes)	Module type (1 byte)	Module SW version (1 byte)	Sampling Time (4 bytes)

0x02	PIR: 0x00~0x09 Piezoelectric: 0x1E~0x27 Power: 0x28~0x3B	0x9999	PIR: 0x01 Piezoelectric: 0x04 Power: 0x05	0x00	0x03E8

**Table:** (b)

10~11	12~13	14~15	16~17	18	19~20	21

Data 0 (2 byte)	Data 1 (2 byte)	Data 2 (2 byte)	Data 3 (2 bytes)	Data 4 (1 byte)	CRC (2 bytes)	ETX (1 byte)

Data (PIR or piezoelectric)	0x00 (null)	0x00 (null)	0x00 (null)	Count	0x0000 (null)	0x03
Data (power: instantaneous power )		Data (power: cumulative power)		

**Table 8:** Module ID registration information of context awareness sensors.

Zone	Location	Activity	Module ID		
			PIR	Piezoelectric	Power
Living room	Entrance	Entering	0 × 00		
	Sofa	Watching TV		0 × 1E	0 × 28
		Reading		0 × 1E	
	Treadmill	Exercising			0 × 29

Bedroom	Entrance	Entering	0 × 01		
	Bed	Reading		0 × 1F	0 × 2A
	Dressing table	Doing makeup		0 × 20	

Study room	Entrance	Entering	0 × 02		
	Reading chair	Reading		0 × 21	

Kitchen	Entrance	Entering	0 × 03		
	Kitchen chair	Dining		0 × 22	
	Cooking table	Meal preparing		0 × 23	0 × 2B
	Sink	Dish-washing		0 × 24	

Bathroom	Entrance	Entering	0 × 04		

**Table 9:** Abstraction process of context information.

Context source	Raw context data	Low-level context information	High-level context information
PIR sensor	0, 01	0, 1	Entering the living room
	0, 00	0, 0	Leaving the living room
	1, 01	1, 1	Entering the bedroom
	1, 00	1, 0	Leaving the bedroom
	2, 01	2, 1	Entering the study room
	2, 00	2, 0	Leaving the study room
	3, 01	3, 1	Entering the kitchen
	3, 00	3, 0	Leaving the kitchen
	4, 01	4, 1	Entering the bathroom
	4, 00	4, 0	Leaving the bathroom

Piezoelectric sensor	30, 3255	30, 997 (g)	Sitting on the sofa in the living room
	30, 176	30, 28 (g)	Not sitting on the sofa in the living room
	31, 3255	31, 997 (g)	Lying on the bed in the bedroom
	31, 176	31, 28 (g)	Not lying on the bed in the bedroom
	32, 3255	32, 997 (g)	Sitting on the chair in the bedroom
	32, 176	32, 28 (g)	Not sitting on the chair in the bedroom
	33, 3255	33, 997 (g)	Sitting on the reading chair in the study room
	33, 176	33, 28 (g)	Not sitting on the reading chair in the study room
	34, 3255	34, 997 (g)	Sitting on the kitchen chair in the kitchen
	34, 176	34, 28 (g)	Not sitting on the kitchen chair in the kitchen
	35, 3255	35, 997 (g)	Standing near the cooking table in the kitchen
	35, 176	35, 28 (g)	Not standing near the cooking table in the kitchen
	36, 3255	36, 997 (g)	Standing near the sink in the kitchen
	36, 176	36, 28 (g)	Not standing near the sink in the kitchen

Power sensor	40, 3172097	40, 937.97 W	Using the TV
	40, 0000	40, 0 W	Turning off the TV
	41, 3164066	41, 929.66 W	Using the treadmill
	41, 0000	41, 0 W	Turning off the treadmill
	42, 030036	42, 30.36 W	Using the desk lamp
	42, 0000	42, 0 W	Turning off the desk lamp
	43, 3156083	43, 921.83 W	Using the microwave
	43, 0000	43, 0 W	Turning off the microwave

**Table 10:** The structure of a transmission data packet for LED control.

0	1	2~3	4	5	6	7	8	9	10~11	12
STX (1 byte)	Module ID (1 byte)	Gateway ID (2 bytes)	Module type (1 byte)	R data (1 byte)	G data (1 byte)	B data (1 byte)	Warm data (1 byte)	Cool data (1 byte)	CRC (2 bytes)	ETX (1 byte)

0x06	0x64~0x96	0x8888	0x02	0x00	0x00	0x00	0x00~0xff	0x00~0xff	0x0000 (null)	0x07

**Table 11:** Result of the lighting control service according to the scenario.